# P-1999. Development and Qualification of IgM and IgG ELISAs for Nipah Virus Detection and Analysis of Human Antibody Response Dynamics

**DOI:** 10.1093/ofid/ofaf695.2163

**Published:** 2026-01-11

**Authors:** Tahsin Tabassum Anonto, Syed Moinuddin Satter, Mohammad Mamun Alam, Sinthia Karim, Ayesha Siddika, Shadman Sakib Choudhury, Wasik Rahman Aquib, Anika Farzin, Dewan Rahman, Sharmin Sultana, Trevor Shoemaker, Michael K Lo, Sayera Banu, Tahmina Shirin, Christina F Spiropoulou, Joel M Montgomery, Mohammed Ziaur Rahman

**Affiliations:** icddr,b, Dhaka, Dhaka, Bangladesh; icddr,b (International Centre for Diarrhoeal Disease Research, Bangladesh), Dhaka, Dhaka, Bangladesh; icddr,b, Dhaka, Dhaka, Bangladesh; icddr,b, Dhaka, Dhaka, Bangladesh; icddr,b (International Centre for Diarrhoeal Disease Research, Bangladesh), Dhaka, Dhaka, Bangladesh; icddr,b, Dhaka, Dhaka, Bangladesh; icddr,b (International Centre for Diarrhoeal Disease Research, Bangladesh), Dhaka, Dhaka, Bangladesh; icddr,b (International Centre for Diarrhoeal Disease Research, Bangladesh), Dhaka, Dhaka, Bangladesh; icddr,b, Dhaka, Dhaka, Bangladesh; Institute of Epidemiology, Disease Control and Research (IEDCR), Dhaka, Dhaka, Bangladesh; Centers for Disease Control and Prevention (CDC), Atlanta, Georgia; US CDC, Atlanta, Georgia; icddr,b (International Centre for Diarrhoeal Disease Research, Bangladesh), Dhaka, Dhaka, Bangladesh; Institute of Epidemiology, Disease Control and Research (IEDCR), Dhaka, Dhaka, Bangladesh; Viral Special Pathogens Branch, Division of High-Consequence Pathogens and Pathology, National Center for Emerging and Zoonotic Infectious Diseases, Centers for Disease Control and Prevention, 1600 Clifton Rd. NE, Atlanta, GA 30333, Georgia, USA., Atlanta, Georgia; Centers for Disease Control and Prevention (CDC), Atlanta, Georgia; icddr,b (International Centre for Diarrhoeal Disease Research, Bangladesh), Dhaka, Dhaka, Bangladesh

## Abstract

**Background:**

Nipah virus (NiV) poses a major public health threat due to severe disease and diagnostic challenges. This study focuses on developing and qualifying enzyme-linked immunosorbent assays ELISAs for detecting NiV-specific IgG and IgM antibodies, supporting retrospective diagnosis, serosurveillance, and vaccine evaluation.

We aimed to develop and qualify in-house indirect ELISA assays for detecting anti-Nipah IgG and IgM antibodies using recombinant glycoprotein G, and to assess the duration of IgM and IgG responses throughout infection and recovery in RT-PCR–confirmed human NiV cases.

**Figure:**
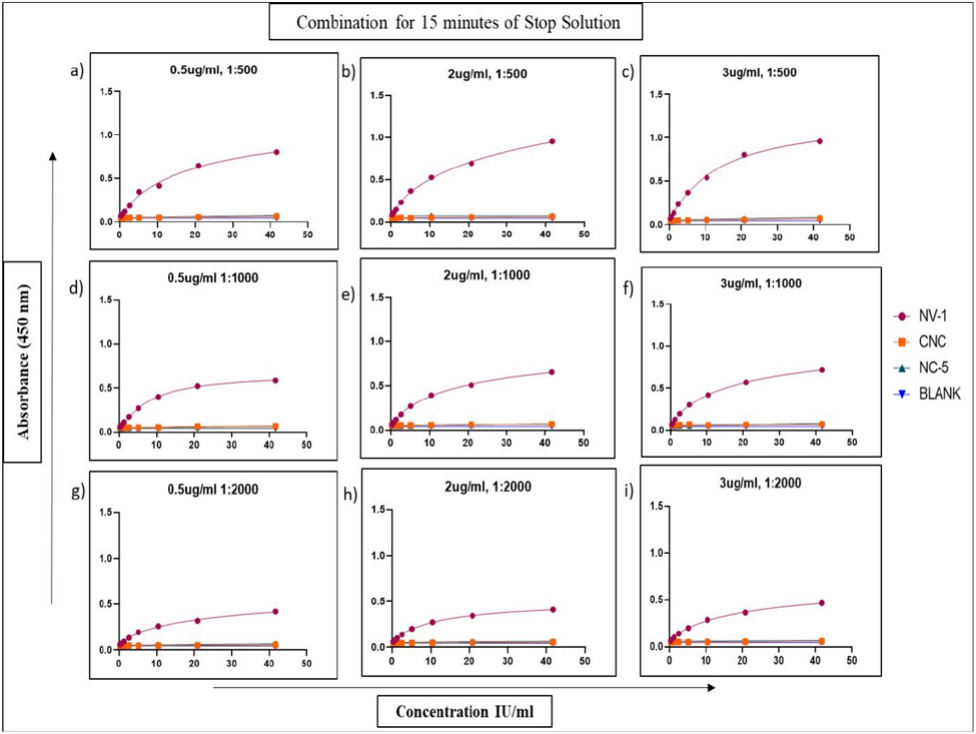
Title: Different concentrations of coating antigen and conjugate with stop solution at 15 minutes combinations a) 0.5 ug/ml coating and 1:500 conjugate dilution b) 2.0 ug/ml coating and 1:500 conjugate dilution c) 3.0 ug/ml coating and 1:500 conjugate dilution d) 0.5 ug/ml coating and 1:1000 conjugate dilution e) 2.0 ug/ml coating and 1:1000 conjugate dilution f) 3.0 ug/ml coating and 1:1000 conjugate dilution g) 0.5 ug/ml coating and 1:2000 conjugate dilution h) 2.0 ug/ml coating and 1:2000 conjugate dilution i) 3.0 ug/ml coating and 1:2000 conjugate dilution. NV-1 (Positive control), CNC (Commercial Negative control), NC-5 (Negative control). Addition of stop solution after 15 minutes of substrate incubation

**Figure:**
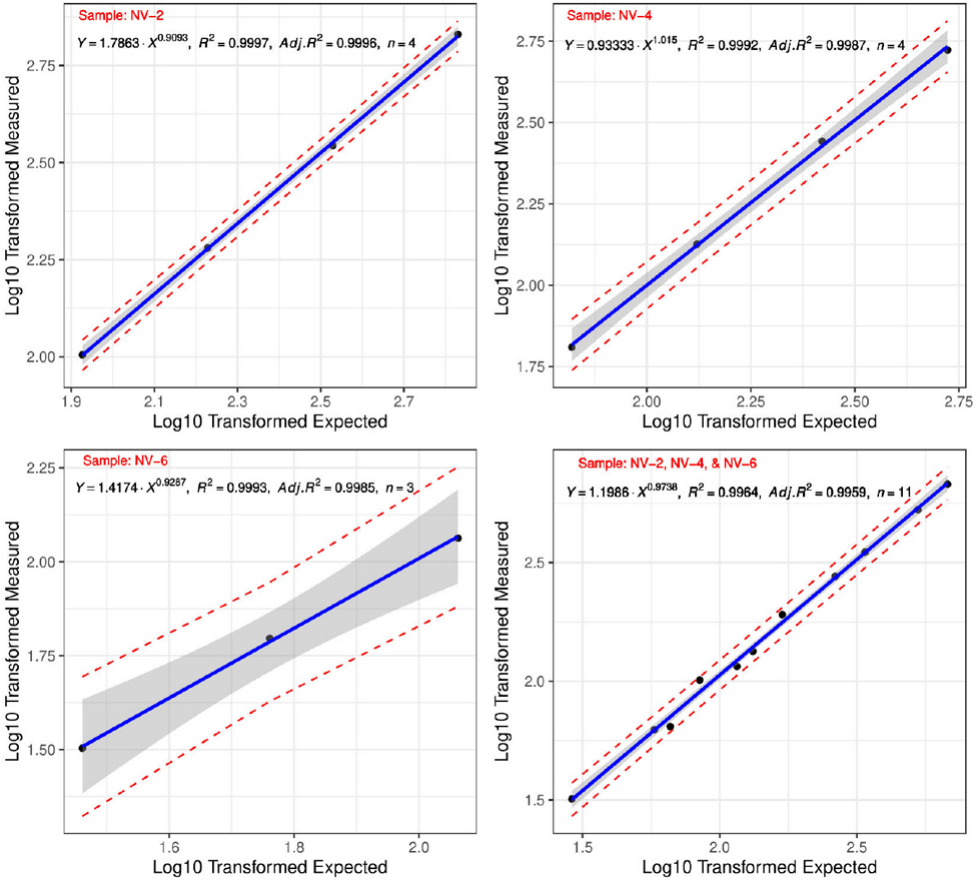
Linear Regression Analysis of Measured vs Expected Log10 Values for Reference Samples Linear regression analysis of log10 transformed measured values versus log10 expected values for three samples (NV-2, NV-4, NV-6). R-squared value closer to 1 suggests a strong linear relationship.

**Methods:**

Indirect ELISAs were developed using recombinant glycoprotein G (Malaysian strain) and evaluated for sensitivity, specificity, precision, linearity, and detection limits using PCR-confirmed positive and negative serum panels. To assess antibody kinetics, serum from 17 RT-PCR–confirmed NiV patients was analyzed over 238 days post-symptom onset. Serial dilutions were performed to quantify antibody titers.

**Figure:**
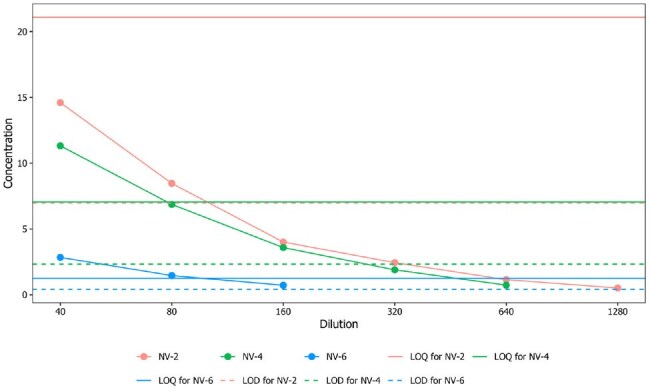
Limits of Detection (LOD) and Quantification (LOQ) The LOD and LOQ results show that for sample NV-2, concentrations below 6.96 IU/ml are considered noise, while concentrations between 6.96 and 21.091 IU/ml can be detected but not reliably quantified. Concentrations above 21.091 IU/ml are accurate for quantification. For sample NV-4, concentrations above 2.328 IU/ml are detectable, and those above 7.055 IU/ml are accurately quantified. For sample NV-6, concentrations above 0.415 IU/ml are detectable, and those above 1.257 IU/ml are quantifiable.

**Figure:**
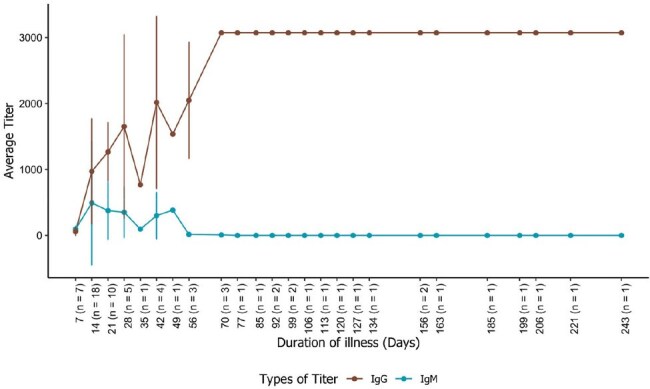
Kinetics of Anti-G IgM and IgG Antibody Responses in Nipah Virus-Infected Patients Mean antibody titers for anti-Nipah Glycoprotein G IgM and IgG were plotted against the duration of illness (in days) to illustrate the temporal dynamics of the humoral immune response in Nipah virus-infected individuals. The data represent averaged values from multiple patient sera tested using an Indirect ELISA. In the graph, brown represents IgG responses, while blue represents IgM responses.

**Results:**

Both IgG and IgM ELISAs demonstrated high accuracy and reproducibility. The IgG ELISA showed 100% sensitivity and 100% specificity. Intra-assay CV ranged from 1.73% to 4.31%, while inter-assay CV was 4.16%. The assay exhibited strong linearity (R² > 0.999) and maintained accuracy within 85%–125 % of expected concentrations. Immunological analysis for antibody response profiling revealed consistent patterns across all cases. IgM responses appeared between Days 3 and 13, peaked around Days 10–21, and declined to undetectable levels by Day 40–54. IgG responses followed shortly after IgM, peaked by Days 27–64, and remained persistently elevated through Days 195–238, indicating durable humoral immunity. These results suggest a short-lived IgM phase followed by durable IgG responses, supporting the use of IgG as a marker of past exposure.

**Conclusion:**

The developed ELISAs are robust tools for NiV antibody detection with applications in diagnostics, surveillance, and vaccine research. Monitoring IgM and IgG kinetics provides insight into infection timelines and immune durability, aiding public health responses in NiV-endemic regions.

**Disclosures:**

All Authors: No reported disclosures

